# Tapetal-Delayed Programmed Cell Death (PCD) and Oxidative Stress-Induced Male Sterility of *Aegilops uniaristata* Cytoplasm in Wheat

**DOI:** 10.3390/ijms19061708

**Published:** 2018-06-08

**Authors:** Zihan Liu, Xiaoyi Shi, Sha Li, Gan Hu, Lingli Zhang, Xiyue Song

**Affiliations:** College of Agronomy, Northwest A&F University, Yangling 712100, Shaanxi, China; liuzihan@nwafu.edu.cn (Z.L.); shixiaoyiaza@163.com (X.S.); lisha2012@nwafu.edu.cn (S.L.); huyunuo@163.com (G.H.); zhanglingli@nwafu.edu.cn (L.Z.)

**Keywords:** cytoplasmic male sterility, gene expression, ROS metabolism, tapetal programmed cell death, wheat (*Triticum aestivum* L.)

## Abstract

Cytoplasmic male sterility (CMS) plays a crucial role in the utilization of hybrid vigor. Pollen development is often accompanied by oxidative metabolism responses and tapetal programmed cell death (PCD), and deficiency in these processes could lead to male sterility. *Aegilops uniaristata* cytoplasmic male sterility (Mu-CMS) wheat is a novel male-sterile line in wheat, which possess important potential in hybrid wheat breeding. However, its CMS mechanisms remain poorly understood. In our study, U87B1-706A, with the *Aegilops uniaristata* cytoplasm, and the maintainer line 706B were used to explore the abortive reason. Compared with 706B, histological analysis and PCD detection of the anther demonstrated that U87B1-706A appeared as delayed tapetal PCD as well as a disorganized organelle phenotype in the early uninucleate stage. Subsequently, a shrunken microspore and disordered exine structure were exhibited in the late uninucleate stage. While the activities of antioxidase increased markedly, the nonenzymatic antioxidant contents declined obviously following overacummulation of reactive oxygen species (ROS) during pollen development in U87B1-706A. Real-time quantitative PCR testified that the transcript levels of the superoxide dismutase (*SOD*), catalase (*CAT*), and ascorbate peroxidase (*APX*) genes, encoding pivotal antioxidant enzymes, were up-regulated in early pollen development. Therefore, we deduce excess ROS as a signal may be related to the increased expression levels of enzyme genes, thereby breaking the antioxidative system balance, resulting in delayed tapetal PCD initiation, which finally led to pollen abortion and male sterility in U87B1-706A. These results provide evidence to further explore the mechanisms of abortive pollen in CMS wheat.

## 1. Introduction

Hybrid wheat is considered as a promising approach to increase yield gains, yield stability and consequently, global productivity of wheat. Cytoplasmic male sterility (CMS) is a maternally inherited trait that results in the failure to produce functional pollen, which is widely employed for hybrid seed production to utilize heterosis in wheat [1]. *Aegilops*, as one of the most successful genera in wheat distant hybridization breeding, plays a critical role in cytoplasmic male-sterile wheat [2]. Tsunewaki et al. [3] demonstrated that *Aegilops uniaristata* (Mu-type) is one of the most valuable cytoplasms for the utilization of wheat male sterility lines in 40 wheat heterogeneous lines except for *Ae. kostchyi*. Besides, *Ae. uniaristata* has many excellent agronomic characteristics and increases resistance to wheat diseases, including stripe rust, leaf rust, stem rust and leaf spot, as well as tolerance to aluminum stress, and can also be used to improve the wheat grain protein content [4]. U87B1-706A, conferred by the cytoplasm from *Ae. uniaristata*, was developed from stable sterile lines by backcrossing with a maintainer over 20 times in Yangling, China. Our previous studies have shown that the line is easier to restore, having the higher germination rate of hybrid seeds as well as powdery mildew resistance compared with other cytoplasmic male sterility lines [5,6]. Therefore, the development of this line is of great value for the breeding and production of hybrid wheat, and it is also an ideal material for research of cytoplasm-nuclear interaction and pollen abortion. However, for economically important U87B1-706A, only agronomic traits have been studied and the abortive mechanism is still not clear and has not been investigated.

In plants, abnormal pollen development is a direct factor causing male sterility. Tapetum, as the innermost sporophytic layer, affects microsporogenesis by supplying proteins, lipids, and pigments, first through secretion and later through degradation. The development of normal pollen requires the proper timing of tapetal degradation, which occurs via developmentally regulated programmed cell death (PCD) [7]. Premature or delayed PCD by tapetal cells disorganizes the supply of the nutrients to microspores, thereby resulting in pollen abortion, which has been described in *Actinidia deliciosa* [8], *Cactaceae* [9], and *Bromeliaceae* [10]. Previous studies suggest this irregular tapetal PCD is tightly controlled by evolutionarily conserved transcriptional cascades [11,12,13]. Recently, the intracellular factors of the transcriptional networks regulating tapetal PCD have been identified, and the researchers indicated that the reactive oxygen species (ROS) act as important signal molecules in tapetal PCD in *Solanaceae*, Arabidopsis and rice [7,14]. However, these mechanisms leading to failed pollen development vary among examples and are rarely well understood. Moreover, few studies have considered the relationship between ROS and natural tapetal PCD in the anthers of CMS wheat, and it is unknown whether the mechanism exists in U87B1-706A wheat.

Mitochondria play a vital role to sustain cellular normal function, which includes initiation of PCD [15], response to oxidative stress signals [16], and synthesis of nucleic acids and proteins [17]. Primarily, the mitochondrion is also an important source of generating ROS, including hydrogen peroxide, superoxide anion radical, and hydroxyl radicals, and it may participate in oxidative damage [18]. If accumulated ROS are not removed from cells effectively and thoroughly, the cells will suffer from a wide variety of adverse effects including damaging nucleic acids, lipids and proteins. Considerable evidence indicates excess ROS accumulation could be associated with the induction of antioxidant genes, thereby causing more efficient enzyme stimulation and protection [19,20]. Plant cells are provided with a high-efficiency antioxidant system consisting of antioxidant enzymes (superoxide dismutase (SOD), catalase (CAT), peroxidase (POD), ascorbate peroxidase (APX), and glutathione peroxidase (GPX)) and non-enzymatic antioxidants (ascorbic acid ASA and glutathione GSH). The alleviation of biotic and abiotic stress in plants transformed with genes, related to antioxidative enzymes, strongly suggests the significantly important role of these enzymes and the genes encoding these enzymes in conferring tolerance [21]. Despite these facts, previous research suggested CMS is linked to the imbalance between production and scavenging of ROS [22]; however, the relationships between dynamic ROS, the antioxidant system, the transcript levels of related genes, and tapetal PCD for CMS remain unclear, especially in wheat.

To deeply explore the abortive mechanisms of the Mu-type CMS in wheat (*Triticum aestivum* L.), in the present study, we investigated the characteristics of U87B1-706A with *Ae. uniaristata* cytoplasm in cytological, physiological, and molecular analyses. In detail, we analyzed morphological and tapetal changes during different developmental stages by histological analysis and further detected the PCD though the terminal deoxynucleotidyl transferase-mediated 2′-Deoxyuridine 5′-Triphosphate nick-end labeling (TUNEL) assay and DNA laddering analysis, where we identified the period of abortion based on iodine-potassium iodide (I_2_-KI) staining, scanning electron microscopy (SEM) observations, and 4′,6-diamidino-2-phenylindole (DAPI) staining. Additionally, we determined physiological indices and validated the expression levels of the *SOD*, *CAT*, and *APX* genes, encoding important antioxidant enzymes by quantitative real-time PCR (qRT-PCR). The results of this study will provide new insights into abortive metabolism in Mu-type CMS wheat, as well as a theoretical basis for the breeding and application of excellent sterile wheat lines.

## 2. Results

### 2.1. Abortive Morphological Features in U87B1-706A

**Figure 1:**
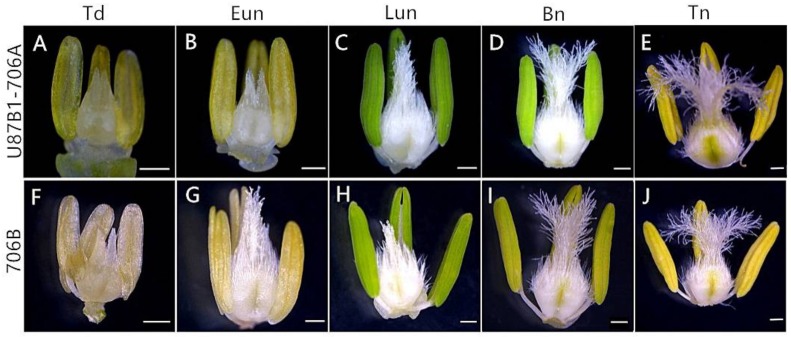
Comparisons of the anther phenotype in U87B1-706A (**A**–**E**) and 706B (**F**–**J**). (**A**,**F**) Td, tetrad stage; (**B**,**G**) Eun, early uninucleate stage; (**C**,**H**) Lun, late uninucleate stage; (**D**,**I**) Bn, binucleate stage; and (**E**,**J**) Tn, trinucleate stage. Scale bars are 500 μm in (**A**–**J**).

**Figure 2:**
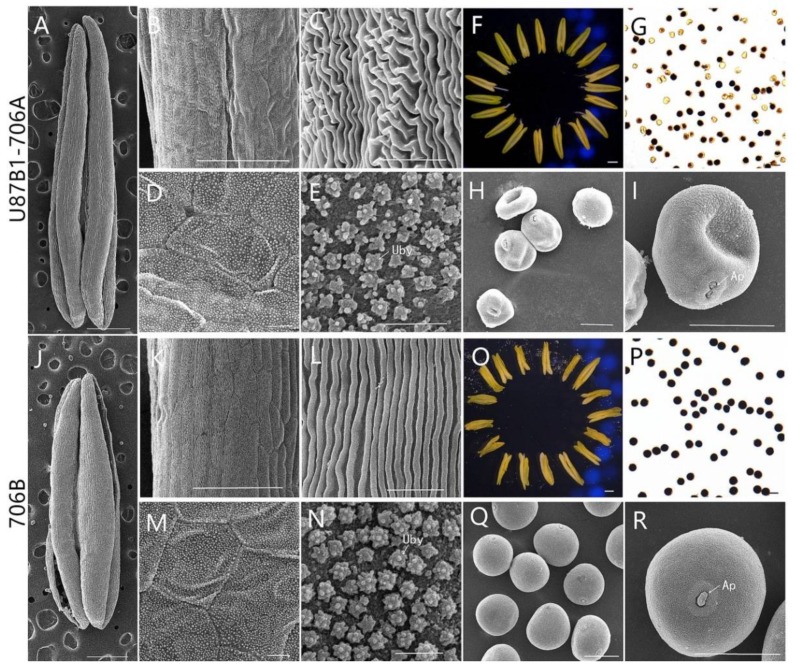
Comparison of scanning electron micrograph observations, I_2_-KI staining, and anther morphology in U87B1-706A (**A**–**I**) and 706B (**J**–**R**) at the trinucleate (Tn) stage. (**A**,**F**,**J**,**O**): anthers; (**B**,**C**,**K**,**L**): outer epidermal cells; (**D**,**E**,**M**,**N**): inner epidermal cells; (**G**,**P**): microspores by I_2_-KI staining; (**H**,**I**,**Q**,**R**): microspores; Uby, Ubisch bodies; Ap, germination aperture. Scale bars are 1 mm in (**A**,**F**,**J**,**Q**); 100 μm in (**B**,**K**); 50 μm in (**G**–**I**,**P**–**R**); 10 μm in (**C**–**E**,**L**–**N**).

In order to gain the abortive morphological features of U87B1-706A, the anthers from five different developmental stages were analyzed in detail. Similar to the anthers of 706B, U87B1-706A appeared to have normal stamens and pistils at the tetrad stage and the early uninucleate stage (Figure 1A,B,F,G). Nevertheless, at the late uninucleate stage and the binucleate stage, compared with the maintainer, the anthers of the sterile line were smaller and more greenish (Figure 1C,D,H,I). Correspondingly, for the anthers of the trinucleate stage, we further used SEM to observe the surfaces of the inner and outer epidermis of anthers. Unlike the fertile plants, the anthers of U87B1-706A were not dehiscent in the trinucleate stage, and no mature pollen grains were released (Figure 1E,J and Figure 2A,F,J,O). The outer epidermal cells of fertile plants anthers were arranged neatly, whereas U87B1-706A displayed shrunken and irregular shapes (Figure 2B,C,K,L). Moreover, compared with 706B, the inner epidermal Ubisch bodies of U87B1-706A were accumulated fewer and more sparsely distributed (Figure 2D,E,M,N and Supplementary Figure S1). Based on observations of microspores at the trinucleate stage, the microspores of 706B were plump and rounded with a gymnotremoid germinal aperture; by contrast, the microspores of U87B1-706A were extremely wizened and atrophied, with a malformed germinal aperture (Figure 2H,I,Q,R). According to I_2_-KI staining and statistical analysis of abortion types, the results showed 34.4% mature pollen grains were typical abortion, which presented an irregular shape and a lack of dye uptake. However, the 65.6% mature microspores were round but the staining was not sufficient, which reflected the trait of stainable abortion. Therefore, unlike 706B, the pollen abortion types of U87B1-706A had typical and stainable abortion [23,24], and the plants were 100% pollen sterile (Figure 2G,P and Supplementary Figure S2). These results demonstrate that nucleo-cytoplasmic interactions have a major influence on the development of the pollen grain and anther.

### 2.2. Tapetal Development in Anthers

**Figure 3:**
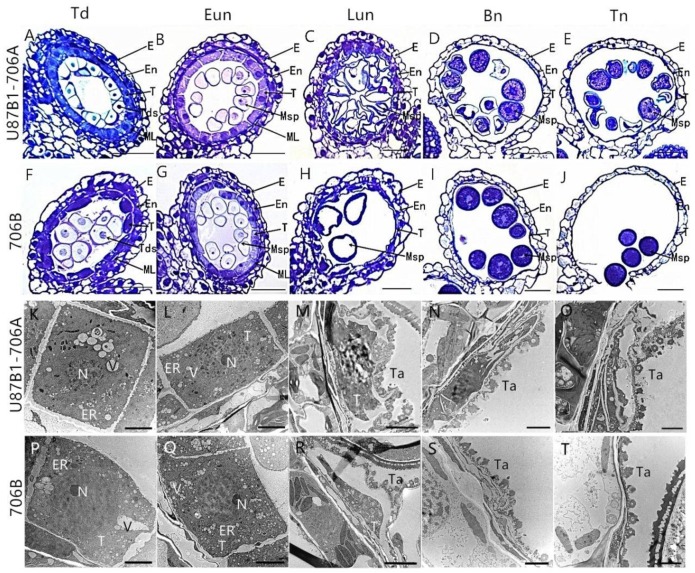
Comparisons of the anther tapetum (**A**–**J**) and ultrastructure (**K**–**T**) in U87B1-706A (**A**–**E**,**K**–**O**) and 706B (**F**–**J**,**P**–**T**) during different developmental stages. (**A**,**F**,**K**,**P**) Td, tetrad stage; (**B**,**G**,**L**,**Q**) Eun, early uninucleate stage; (**C**,**H**,**M**,**R**) Lun, late uninucleate stage; (**D**,**I**,**N**,**S**) Bn, binucleate stage; and (**E**,**J**,**O**,**T**) Tn, trinucleate stage. E, epidermis; En, endothecium; ER, endoplasmic reticulum; ML, middle layer; Msp, microspores; N, nucleus; T, tapetum; Ta: tapetosome; Tds, tetrads; V: vacuole. Scale bars are 50 μm in (**A**–**J**) and 2 μm in (**K**–**T**).

To further explore the reason of pollen and anther abortion in U87B1-706A, we first conducted observations of the tapetum during the different developmental stages based on paraffin and semi-thin transverse sections and CellSens Entry software. At the tetrad stage and early uninucleate stage, light microscopy observations revealed that the anther locules were composed of four layers, from inside to outside, which were the innermost tapetum, the middle layer, the endothecium, and the epidermis. In addition, there were no obvious differences at the tetrad stage and early uninucleate stage in the tapetal cells between 706B and U87B1-706A, which could not be degraded (Figure 3A,B,F,G, Supplementary Figures S3A,B,F,G and S4). However, during the later uninucleate stage, the areas of the tapetal cells in U87B1-706A were unconventionally larger than those in 706B, and they occupied the majority of the anther locule instead of degenerating to secrete nutrients for microspore formation (Figure 3C,H, Supplementary Figures S3C,H and S4). At the binucleate stage, the tapetum shrank and condensed obviously and was reduced to a very thin layer (Figure 3D,I and Supplementary Figure S3D,I). Up to the trinucleate stage, the outlines of the tapetal cell in 706B were completely invisible and the anther wall layers were thinner, thereby contributing to the release of mature pollen grains by anther dehiscence to pollinate female gametophytes. In addition, the epidermis and endothecium of sterile and fertile anthers in the trinucleate stage were thicker compared with the binucleate stage (Figure 3J and Supplementary Figure S3J). However, the anther wall of U87B1-706A was not fractured at the trinucleate stage, and the invasion of the cell mass could cause the abortion of microspores (Figure 3E and Supplementary Figure S3E). Therefore, we deduced that delayed tapetum dysfunction may result in the abortion of U87B1-706A.

To verify the inference of the paraffin and semithin sections and further observe the tapetal organelles, transmission electron microscopy (TEM) was implemented during all of the developmental stages. Consistent with the results of semi-thin sections, the results of the ultrathin section confirmed the abnormal degradation of the tapetum also occurs in the later uninucleate stage. The tapetal cells were still quite intact at the tetrad and early uninucleate stages in U87B1-706A, whereas nuclear chromatins were more blurred and the nuclear membranes were less obvious than those in 706B. The tapetal cells exhibited a spongy feature because of the distribution of numerous vesicles and secretory vacuoles, and endoplasmic reticula expanded continually in the anther locules of U87B1-706A (Figure 3K,L,P,Q). From the later uninucleate stage to trinucleate stage, compared with 706B, tapetosomes of U87B1-706A were comparatively scattered and displayed distinct abnormality in tapetal cells, and the organelles were also indistinct (Figure 3M–O,R–T). These observations indicate that the abortive reason is probably anomalous changes of organelles in tapetum, thereby causing the delayed tapetal degradation. 

### 2.3. Tapetal PCD Detection in Anthers

**Figure 4:**
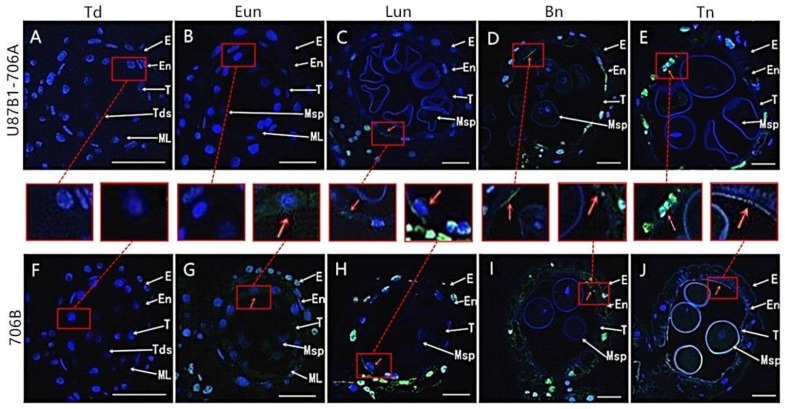
TUNEL assays to detect anther tapetum PCD in U87B1-706A (**A**–**E**) and 706B (**F**–**J**) during different developmental stages. (**A**,**F**) Td, tetrad stage; (**B**,**G**) Eun, early uninucleate stage; (**C**,**H**) Lun, late uninucleate stage; (**D**,**I**) Bn, binucleate stage; and (**E**,**J**) Tn, trinucleate stage. E, epidermis; En, endothecium; ML, middle layer; T, tapetum; Tds, tetrads; Msp, microspores. The green fluorescence denoted by the red arrows indicates nuclei with TUNEL-positive staining. To make green fluorescence visible, the red square shows enlarged example. Scale bars are 50 μm in (**A**–**J**).

In order to further characterize the tapetal PCD, we analyzed the cleavage of nuclear DNA using the TUNEL assay in U87B1-706A and 706B during different developmental stages. At the tetrad stage, there was no TUNEL fluorescence signal in tapetal cells of U87B1-706A and 706B (Figure 4A,F). At the early uninucleate stage, green TUNEL-positive signals were found in 706B tapetum, thereby indicating that the PCD of the tapetum was present in this stage, while the TUNEL PCD signals were not detected in the tapetum of U87B1-706A (Figure 4B,G and Supplementary Figure S5). Subsequently, until the later uninucleate stage, a weaker TUNEL-positive signal was produced in U87B1-706A tapetum. Compared with U87B1-706A, stronger TUNEL-positive signals occurred in the degenerating tapetum in 706B, which explained the obvious accumulation of DNA cleavage in this stage (Figure 4C,H and Supplementary Figure S5). From the binucleate stage to the trinucleate stage, the intense TUNEL-positive signals were also observed in the epidermis and the endothecium cells in U87B1-706A and 706B (Figure 4D,E,I,J). In a word, these observations verified that the initiation of tapetal cell PCD was delayed in U87B1-706A.

For both U87B1-706A and 706B, DNA damage levels were determined during different developmental stages in the anthers by DNA laddering to better determine the TUNEL assay result. Similar to the TUNEL PCD detection, a significant DNA fragmentation in 706B first appeared at the early uninucleate stage, whereas the DNA fragmentation of U87B1-706A formed ladders with bands at the later uninucleate stage (Supplementary Figure S6). The results of the DNA ladder assays indicate that the anther tapetum exhibited typical PCD characteristics, where U87B1-706A exhibited deferred PCD in the tapetum. We also found that the molecular features of PCD were observed earlier than the cytological phenotype in U87B1-706A and 706B.

### 2.4. Cytological Characteristics of Microspores

**Figure 5:**
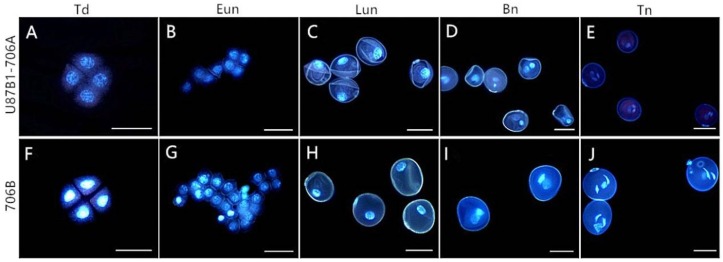
DAPI staining of microspores in U87B1-706A (**A**–**E**) and 706B (**F**–**J**). (**A**,**F**) Td, tetrad stage; (**B**,**G**) Eun, early uninucleate stage; (**C**,**H**) Lun, late uninucleate stage; (**D**,**I**) Bn, binucleate stage; and (**E**,**J**) Tn, trinucleate stage. Scale bars are 50 μm in (**A**–**J**).

In plants, the tapetum can provide the nutrients for the normal development of microspores though timely tapetal PCD [25]. Thus, the DAPI and acetocarmine staining were carried out to investigate pollen development to further clarify whether the abnormal pollen development was associated with abnormal tapetal PCD in U87B1-706A. At the tetrad stage and the early uninucleate stage, no obvious variations in microspore development of U87B1-706A were observed compared with 706B: the microspores were released from the tetrads and experienced normal development, where the nuclei were located at the center of the cell (Figure 5A,B,F,G and Supplementary Figure S7). At the late uninucleate stage, the microspores of 706B and U87B1-706A had a huge vacuole, where the nuclei were displaced to the opposite side of the germination aperture. However, unlike 706B, the microspores were severely corrugated in U87B1-706A in the late uninucleate stage (Figure 5C,H and Supplementary Figure S7C,H). Up to the binucleate stage, the microspores of 706B gradually enlarged, thereby forming rounded and compact vegetative and sperm nuclei in 706B. In contrast to 706B, the vegetative and sperm nuclei were slightly bigger in U87B1-706A than in 706B, and some of the vegetative nuclei were not clear (Figure 5D,I and Supplementary Figure S7D,I). At the trinucleate stage, the sperm nuclei became round in U87B1-706A instead of the normal spindle shape (Figure 5E,J and Supplementary Figure S7E,J). Therefore, we suggest that the delayed tapetal PCD of U87B1-706A led to abnormal pollen development in the late uninucleate stage due to not contributing to nutrients in time for the development of microspores.

**Figure 6:**
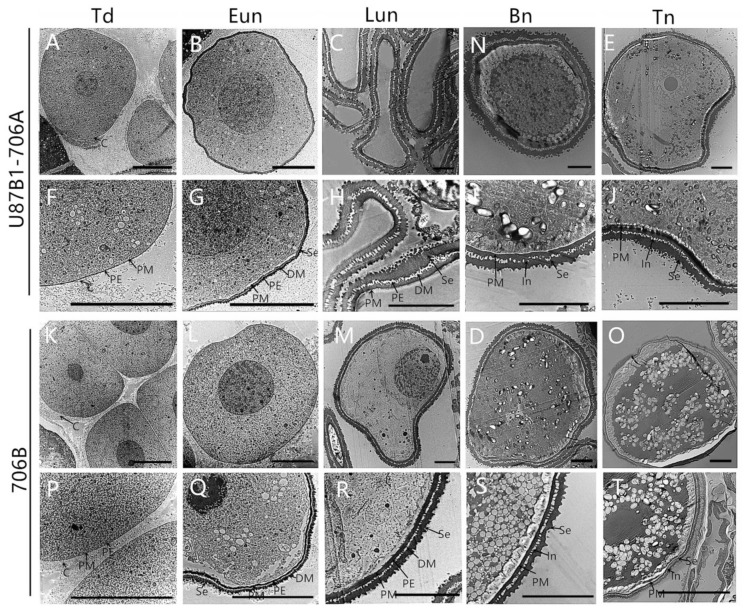
Transmission electron micrographs of the microspores in U87B1-706A (**A**–**J**) and 706B (**K**–**T**). (**A**,**F**,**K**,**P**) Td, tetrad stage; (**B**,**G**,**L**,**Q**) Eun, early uninucleate stage; (**C**,**H**,**M**,**R**) Lun, late uninucleate stage; (**D**,**I**,**N**,**S**) Bn, binucleate stage; and (**E**,**J**,**O**,**T**) Tn, trinucleate stage. C, callose; PE, primexine; Se, sexine; DM, deposit materials; PM, plasma membrane; In, intine. Scale bars are 3 μm in (**A**–**T**).

During the pollen development process, proper tapetal PCD also could provide enough sporopollenin to maintain normal pollen wall development [26]. Therefore, we used TEM to observe the ultrastructure of microspores to further confirm whether the abnormal pollen wall was synchronized with tapetal PCD delay in U87B1-706A. During the tetrad stage, the pollen wall is composed of plasma membrane, primexine and callose. Up to the early uninucleate stage, the sexine was formed with the continuous accumulation of sporopollenins (deposit materials). In these two stages, there were no distinct differences between U87B1-706A and 706B in the pollen wall (Figure 6A,B,F,G,K,L,P,Q). However, unlike 706B, the abnormal accumulation of sporopollenin in U87B1-706A resulted in the inability to form normal sexine in the subsequent process of pollen development. Disordered and incomplete primary exine and sexine structures appeared on U87B1-706A microspore surface (Figure 6C–E,H–J,M–O,R–T). These observations indicate that incompatible nucleus and mitochondria might trigger delayed PCD in the tapetum, thereby hindering the secretion of sporopollenin during pollen development, which causes defective pollen exine, and results in male sterility of U87B1-706A.

### 2.5. O_2_^−^ Generation Rate and Contents of H_2_O_2_ and MDA

**Figure 7:**
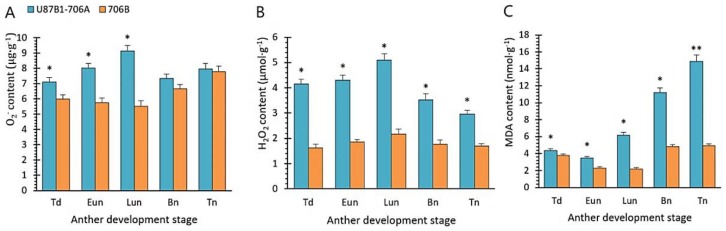
Accumulation of O_2_^–^, H_2_O_2_, and MDA in the anthers of U87B1-706A and 706B during various developmental stages. Td, tetrad stage; Eun, early uninucleate stage; Lun, late uninucleate stage; Bn, binucleate stage; and Tn, trinucleate stage. (**A**) O_2_^–^ contents; (**B**) H_2_O_2_ contents; (**C**) MDA contents. Students’ *t* test * *p* < 0.05, ** *p* < 0.01. Each value represents the mean ± SD (*n* = 3).

To further explore the reason for abnormal anther tapetal PCD and microspores, we examined the O_2_^−^, H_2_O_2_ and malondialdehyde (MDA) contents during the different developmental stages in the anthers of U87B1-706A and 706B. The O_2_^−^ and H_2_O_2_ contents were continuously increased in U87B1-706A with peak values in the late uninucleate stage, which were significantly higher than in 706B during the early development of pollen (Figure 7A,B). The later stages of pollen development were accompanied by the extremely high MDA contents in U87B1-706A than in 706B, whereas the MDA contents were low in the tetrad stage and early uninucleate stage (Figure 7C). Thus, we suggest that the excessive accumulation of ROS in U87B1-706A during the early anther developmental stages could act as a signal to trigger the anomalous tapetal PCD.

### 2.6. Activities of Antioxidant Enzymes and Nonenzymatic Antioxidants

**Figure 8:**
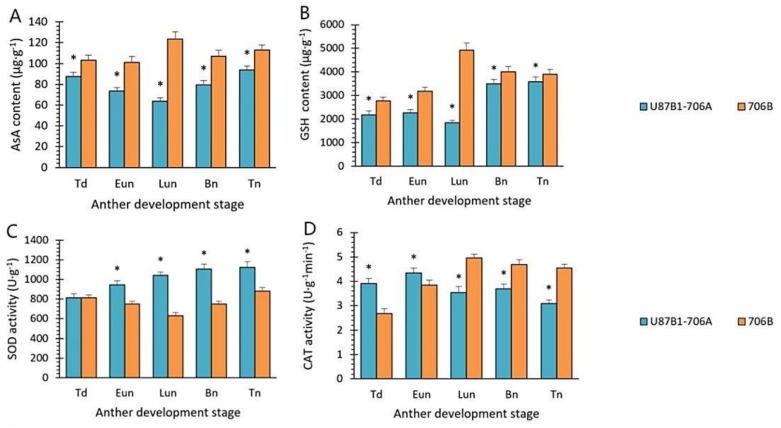

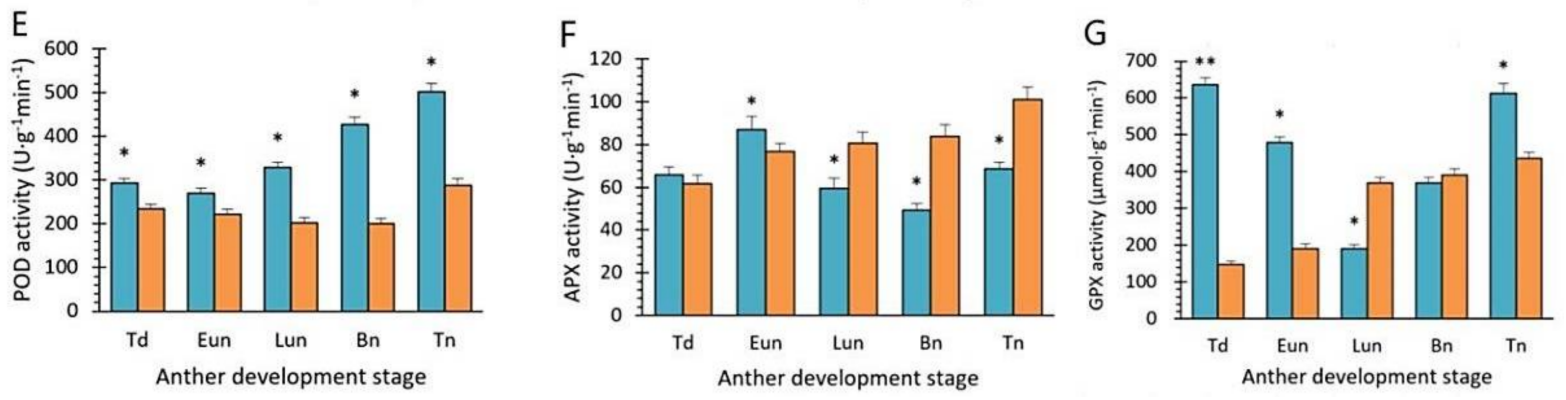
Nonenzymatic antioxidant contents (**A**,**B**) and activities of antioxidant enzymes (**C**–**G**) in anthers from U87B1-706A and 706B during different developmental stages. Td, tetrad stage; Eun, early uninucleate stage; Lun, late uninucleate stage; Bn, binucleate stage; and Tn, trinucleate stage. (**A**) AsA contents; (**B**) GSH contents; (**C**) SOD activity; (**D**) CAT activity; (**E**) POD activity; (**F**) APX activity; (**G**) GPX activity. Students’ *t* test * *p* < 0.05, ** *p* < 0.01. Each value represents the mean ± SD (*n* = 3).

As compared to 706B, there was a decline in the ASA and GSH activity levels in U87B1-706A during the entire period of pollen development, where the differences between U87B1-706A and 706B were significant. In addition, contrary to 706B, the ASA and GSH activity levels had a rapid decline until the late uninucleate stage in U87B1-706A, before an increase in the later stages of pollen development (Figure 8A,B). Furthermore, the activities of antioxidant enzymes, i.e., SOD, CAT, POD, APX, and GPX, were determined. The SOD and POD activities were always higher in U87B1-706A than those in 706B during the whole course of pollen development, while the CAT, APX contents were higher than 706B only in the tetrad stage and the early uninucleate stage. Likewise, a sharp increase in the GPX contents occurred in U87B1-706A during the tetrad stage; differences were significant between 706B and U87B1-706A (*p* < 0.01) (Figure 8C–G). According to these outcomes, it is possible that the nonenzymatic antioxidants may have been weaker in U87B1-706A than 706B, thereby causing excess accumulation of ROS, thus leading to the up-regulation of the activities of antioxidant enzymes, finally disrupting the balance of antioxidant system and endogenous hormones.

### 2.7. Expression Levels of Antioxidant Enzyme Genes

**Figure 9:**
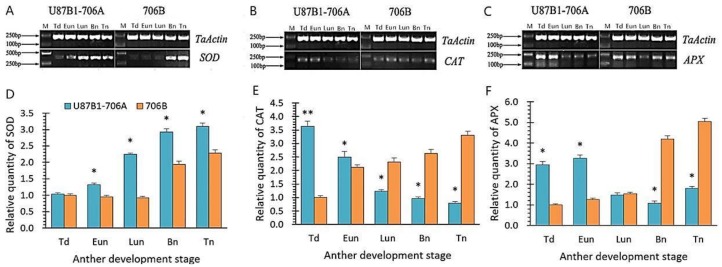
Results of gene expression for *SOD* (**A**,**D**), *CAT* (**B**,**E**), and *APX* (**C**,**F**) in anthers from U87B1-706A and 706B during different developmental stages. Td, tetrad stage; Eun, early uninucleate stage; Lun, late uninucleate stage; Bn, binucleate stage; and Tn, trinucleate stage. M, Marker D2000. Students’ *t* test * *p* < 0.05, ** *p* < 0.01. Each value represents the mean ± SD (*n* = 3).

The expression levels of *SOD*, *CAT* and *APX* genes encoding important antioxidant enzymes were determined in U87B1-706A and 706B during the different developmental stages. In addition, the correlations between the enzyme activities and genes expression levels were analyzed through the tendency chart and SPSS software. The dynamics of the *SOD* gene expression levels were similar between U87B1-706A and 706B, but higher than that of 706B during all anther developmental stages (Figure 9A and Supplementary Figure S8). Additionally, the correlative assay showed that SOD enzyme activities and *SOD* gene expression levels were extremely significantly correlated in U87B1-706A (Supplementary Figure S9). The *CAT* gene and *APX* expression levels continuously decreased in U87B1-706A, whereas those of 706B gradually increased throughout anther development. Moreover, there was a significant difference between U87B1-706A and 706B in the *CAT* gene expression level at the tetrad stage (Figure 9B,C and Supplementary Figure S8). The results of correlations indicated that all of the enzyme activities and the related gene expression levels were positively correlated (Supplementary Figure S9). From these results, it can be concluded that the disordered dynamics of ROS, the expression of related enzymes, and the antioxidant system were consistent with the abnormal tapetal PCD in the anther of U87B1-706A.

## 3. Discussion

### 3.1. The Critical Period of Abortion in Mu Type-CMS

In angiosperms, normal release of pollen from the anther and proper development are essential for successful reproduction. However, many studies have shown that different plants vary in terms of their abortion periods and characteristics. Previous research suggests that 4% of abortions occur in the early stages of meiosis, 57% during the tetrad stage, and 39% in the microspore developmental stage in monocotyledonous plants. In dicotyledonous plants, 27% of abortions occur in early meiosis, 58% in the tetrad stage, and 15% in the microspore developmental stage [27]. According to our light and electron microscopy analyses of the cytological study, we discovered that the microspores of U87B1-706A first showed abortive characteristics at the late uninucleate stage, where they eventually were completely aborted by the trinucleate stage. Tapetum degradation is considered as a direct factor of pollen abortion in male sterility [28,29]. At the same time, compared with the control plants 706B, there was an obviously delayed degradation of tapetal cell and abnormalities of organelles of U87B1-706A at the late uninucleate stage. Generally speaking, morphological features of the degradation of the tapetum are identified as results of PCD during the development of the anther [9,30]. When PCD occurs in the tapetum, normal DNA could degrade into nuclear DNA fragmentation to form nucleosomes of more than 140 bp, and subsequently abnormal cytological morphology will be visible [31,32]. Furthermore, in this study, the TUNEL and DNA laddering were performed to precisely explore the accurate stage of pollen abortion, and the results indicated that the TUNEL PCD signals of 706B was first present in the early uninucleate stage, while there were no TUNEL PCD signals in U87B1-706A. Therefore, we deduce the critical period of abortion of U87B1-706A is probably at the early uninucleate stage owing to the delayed initiation of tapetal PCD.

### 3.2. Tapetal PCD and Pollen Abortion in Mu Type-CMS

In the present study, based on our morphological observation, four anther cell wall layers appeared, which successively were the epidermis, the endothecium, the middle layer, and the tapetum from exterior to interior. The tapetum is a very special cell layer between the anther wall and microspores, which is located in the innermost of anther wall and secretes nutrients, sporopollenin precursors, and enzymes for pollen development [33]. Tapetal degeneration is a very strict process that will lead to pollen abortion if it is not performed in an orderly and synchronized manner [34]. In this study, our cytological semi-thin section combined with the ultrathin section analyses indicated although the tapetal phenotype of U87B1-706A appeared normal based on semi-thin sections at the early uninucleate stage, the tapetal organelles were initially abnormal and presented an irregular appearance in this stage, which demonstrates that organelle disorder occurred first in the abnormal degradation of the tapetum. In general, tapetal degradation is regarded as the result of PCD, which is resulted of DNA fragmentation [35]. Similarly, TUNEL and DNA laddering analysis provided direct evidence that the PCD of tapetal cells was also detected in 706B and U87B1-706A, and further demonstrated that U87B1-706A underwent deferred PCD of the tapetal cells, thereby causing the collapse of microspores and male sterility due to a lack of essential materials and signals for proper microspore development. How does the abnormal PCD of the tapetum cause pollen abortion? Previously, researchers suggested that tapetal PCD could provide the completion of the extracellular sculpting of the pollen and promote metabolites, pollen wall synthesis and pollen deposition [7]. Therefore, differences in biosynthesis and exogenous inputs in sterile plants disrupted energy metabolism, thus accelerating pollen abortion [26]. The pollen exine, as the outermost layer of the pollen wall, is composed mostly of sporopollenin, which is secreted from the tapetum via PCD [36]. In U87B1-706A, the delayed tapetal PCD could lead to incomplete sporopollenin synthesis, thereby causing irregular microspore morphology and male sterility.

### 3.3. ROS and Antioxidant Defense System in Mu Type-CMS

Plant mitochondria are a main cellular source of ROS throughout the entire respiratory process, and are one of the major targets of ROS under oxidative stress, which can disrupt mitochondrial normal function due to excessive ROS production [37]. In our studies, U87B1-706A revealed a continuous increase in O_2_^−^ and H_2_O_2_ contents from the tetrad stage to late uninucleate stage, which is the most vital stage of microspore abortion, with maximum values in the late uninucleate stage. Simultaneously, the ROS contents of U87B1-706A remained higher than those of 706B during the whole anther developmental course [38]. MDA, as the most common indicator of lipid peroxidation, is often associated with oxidative stress. Once formed, such high MDA can lead to cell damage through nucleic acid reactions with proteins, lipids and organelles [39]. The present study revealed that there is an obvious increase in MDA contents in U87B1-706A during the whole pollen developmental process, which were always higher than those in 706B along with the over-accumulation of ROS. The reason for the superfluous accumulation of O_2_^−^, H_2_O_2_ and MDA contents in male sterility could be related to the activities of antioxidative enzymes and nonenzymatic antioxidant components in response to the expression levels of relevant genes. Additionally, this result also indicates that the amplitude of dynamic ROS during all anther development stages is closely related to the occurrence and development of tapetum PCD. To better resist oxidative stress, plants form a high-efficiency antioxidant defensive system in the cell and thereby maintain the normal generation and clearance rates of ROS to reduce their effects on various biological molecules [40]. SOD, POD, CAT APX, and GPX are identified as key antioxidative enzymes that can eliminate redundant ROS [18]. Moreover, ASA and GSH as well as the antioxidative enzymes can constitute an ASA-GSH cycle system that effectively scavenges free radicals [41]. Our results showed SOD and POD activities in U87B1-706A were invariably higher than those in 706B during the whole anther developmental process to defend against excess ROS accumulation. However, contrary to 706B, CAT, APX, GPX activities levels decreased in U87B1-706A at the late pollen development. Analogously, compared with 706B, the non-enzymatic antioxidant contents were always lower in U87B1-706A. It is possible that the excessive generation of ROS destroyed the normal antioxidant system, leading to a decrease in enzyme activity, thereby breaking the balance of the endogenous IAA pool. Many studies suggested that the over-accumulation of ROS can also affect the expression of various genes by oxidative damage [42,43]. Moreover, previous studies on the mitochondrial genome have revealed some genes or open reading frames (ORFs) are often chimeric and co-transcribed with genes that encode the mitochondrial subunits of the respiratory enzymes related to ROS release and further indicated that mutation and recombination have a direct relationship with CMS [20,44,45]. In our study, we performed RT-PCR to determine the expression levels of the genes encoding important antioxidant enzymes. The results showed the expression levels of the *SOD*, *CAT* and *APX* genes were uniformly up-regulated during the early stages of pollen development in U87B1-706A, which were similar to the enzyme activity trends. It is possible that the disorganized transcript levels of enzymes genes are tightly linked to ROS generation, and ROS overproduction is not effectively eradicated by the antioxidative system, which result in the microspores chronically suffering from oxidative stress during anther development. In brief, we provide evidence supporting a vital role of the encoding of important antioxidant enzymes genes in dynamic tapetal ROS during the anther developmental stages.

## 4. Methods

### 4.1. Plant Materials

Cytoplasmic male sterile line, U87B1-706A containing *Ae. uniaristata* cytoplasm, and the maintainer line, 706B developed by Norwest A&F University, which had the same nuclear background and which belonged to facultative wheat (*Triticum aestivum* L.) were used in our study. U87B1-706A was developed from a stable sterile line by backcrossing with 706B over 20 times [46]. All materials were planted conventionally at the Northwest A&F University experimental farm in Yangling (108° E, 34°15′ N), China, during October 2014. All plants were arranged in randomized complete block designs with three replications. To identify the stability of sterility, U87B1-706A was checked by bagging in April 2015. The results demonstrated that the self-setting rate of U87B1-706A was zero, and thus the male sterility was complete and stable [6]. The dates of the microspore developmental stages were recorded for the sterile line U87B1-706A and the maintainer line 706B as described previously [31].

### 4.2. Phenotypic Characterization

According to the previous classification of microsporogenesis, we divided the overall anther development period into five stages: tetrad stage (Td), early uninucleate period (Eun), late uninucleate period (Lun), binucleate stage (Bn), and trinucleate stage (Tn) [47]. Photographs of anthers were obtained from the five microspore developmental stages using a stereomicroscope (Motic, K4000, Hong Kong, China). For scanning electron microscopy, anthers and microspores of trinucleate stage were dehydrated in a series of ethanol solutions, then dried, and observed by a scanning electron microscope (JSM-6360LV, JEOL, Tokyo, Japan) [48]. To identify the fertility of U87B1-706A and 706B, mature pollen grains from dehiscing anthers were stained using I_2_-KI. In addition, we counted the I_2_-KI staining abortive ratio and the numbers of Ubisch bodies using IPP 6.0 software (Media Cybernetics, Rockville, MD, USA).

### 4.3. Histological Analysis

For observation of paraffin sections, anthers of different developmental stages were substituted by xylene and embedded in paraffin wax and cut into 8 μm transverse sections and stained with 0.2% toluidine blue. For semithin sections as well as transmission electron microscopy observation, anthers were fixed, embedded, and stained as described by Zhang et al. [49]. The paraffin and semithin sections were photographed using a DS-U2 high resolution camera mounted on a microscope (Nikon, ECLIPSE, E600, Tokyo, Japan) and processed with NIS-Elements software (Nikon, Tokyo, Japan), and the ultrathin sections were observed and obtained with transmission electron microscope (Hitachi, H-7650, Tokyo, Japan) and an 832 charge-coupled device camera (Gatan, Abingdon, VA, USA). Then, we calculated the area of the tapetum cells in the section using CellSens Entry software (Olympus, Tokyo, Japan). The morphology and nuclear DNA of the microspores from various developmental stages were observed by stained with 1% acetocarmine and by staining the nuclei with DAPI.

### 4.4. TUNEL and DNA Laddering Analysis

For the TUNEL assay, paraffin sections were dehydrated and washed in a graded ethanol series, before incubating with 20 μmL^–1^ Proteinase K (Roche, Basel, Switzerland) for 15 min, and then washed in phosphate-buffered saline (PBS; pH = 7.4) for 15 min. In situ nick-end labeling of nuclear DNA fragmentation was performed with the In Situ Cell Death Detection Kit, POD (Roche), following the manufacturer’s instructions. Samples were analyzed with a fluorescence confocal scanner microscope (Nikon, Tokyo, Japan) using a 450/515 nm excitation/emission spectrum for TUNEL fluorescein and a 358/461 nm excitation/emission spectrum for DAPI as described in [15]. For DNA laddering analysis, total DNA was isolated from the anthers in different developmental stages [50]. Then, the total DNA was redissolved in Tris-EDTA (10 mmol L^−1^ Tris-HCl (pH 8.0), 5 mmol L^−1^ EDTA) and incubated at 37 °C for 60 min in the presence of RNase A (100 g mL^−1^). Subsequently, 10 μg of DNA was separated by electrophoresis on a 1.8% (*w*/*v*) TBE-agarose gel. The gel was then stained with ethidium bromide to visualize the DNA ladder [16].

### 4.5. Quantification of ROS and Antioxidants

Anthers (1 g) from each developmental stage were collected to determine the physiological indexes. The rate of superoxide anion (O_2_^−^) production, the H_2_O_2_ contents, and the MDA contents were measured according to Ba et al. [13]. The activities of antioxidative enzymes (SOD, POD, CAT, APX and GPX) were measured as described by Bibi et al. [51]. The AsA contents were calculated according to Jiang and Zhang [52]. The GSH contents were determined according to the method of Wang et al. [53]. All reactions were performed with three replications for each material from all developmental stages.

### 4.6. Reverse Transcription-PCR (RT-PCR) and Quantitative Real-Time-PCR (qRT-PCR) Analysis of Antioxidant Enzyme Genes

Total RNA was extracted using TRIZOL reagent (Tiangen, Beijing, China), and quantified spectrophotometrically by measuring absorbance at 260 nm. Reverse transcription polymerase chain reaction (RT-PCR) and quantitative real-time-PCR (qRT-PCR) were used to study the expression of genes (*SOD*, *CAT*, *APX*). Primers used in this article were obtained from previously reported papers (Supplementary Table S1). For qRT-PCR, we used HiScript^TM^ Q Select RT SuperMix for qPCR (Vazyme, Nanjing, China) to synthesize cDNA and we used AceQ^®^ qPCR SYBR^®^ Green Master Mix (Vazyme, China) to analyze qRT-PCR reaction products in ABI PRISM 7000 Sequence Detection System (Thermo Fisher, Waltham, MA, USA). For RT-PCR, the temperature profile was as follows: 95 °C for 3 min, followed by 35 cycles at 95 °C for 30 s, 56 °C for 30 s, and 72 °C for 15 s. All of the experiments were performed in triplicate.

### 4.7. Statistical Analysis

Statistical analyses were performed during different anther stages for each experiment (I_2_-KI staining, counts of Ubisch bodies, area of the tapetum cells, determination of physiological indexes, and qRT-PCR) using one-way analysis of variance. Significant differences and correlation analysis were evaluated using SPSS statistical software (IBM, New York, NY, USA) and Excel Office (Microsoft Corporation, Washington, DC, USA). Data are the mean ± SD of three biological replicates.

## 5. Conclusions

Based on cell biology, physiology, and molecular biology, we believe that excess ROS may be related to up-regulated transcript levels of *SOD*, *CAT*, *APX* genes, and may result in the destruction of the antioxidant system equilibration, thereby triggering the delayed tapetal PCD in U87B1-706A, which ultimately led to pollen abortion. These results provide a necessary theoretical basis for further study of the interaction between the ROS production system and tapetum transcription network in pollen abortion.

## Supplementary Materials

Supplementary materials can be found at http://www.mdpi.com/1422-0067/19/6/1708/s1.

## Abbreviations

CMS	cytoplasmic male sterility
PCD	programmed cell death
ROS	reactive oxygen species
SOD	superoxide dismutase
CAT	catalase
POD	peroxidase
APX	ascorbate peroxidase
GPX	glutathione peroxidase
ASA	ascorbic acid
GSH	glutathione
TUNEL	terminal deoxynucleotidyl transferase-mediated dUTP nick-end labeling
SEM	scanning electron microscopy
DAPI	4′,6-diamidino-2-phenylindole
TEM	transmission electron microscopy
MDA	malondialdehyde
Uby	Ubisch bodies
Ap	germination aperture
E	epidermis
En	endothecium
ML	middle layer
T	tapetum
Tds	tetrads
Msp	microspores
ER	endoplasmic reticulum
N	nucleus
Ta	tapetosome
V	vacuole
PE	primexine
Se	sexine
DM	deposit materials
PM	plasma membrane
In	intine
