# Supplementary material for: Tapetal-Delayed Programmed Cell Death (PCD) and Oxidative Stress-Induced Male Sterility of Aegilops uniaristata Cytoplasm in Wheat
Source: Int J Mol Sci. 2018 Jun 8;19(6):1708. doi: 10.3390/ijms19061708 (PMC6032135; doi:10.3390/ijms19061708)

Supplementary data

Supplementary Figure S1. Numbers of Ubisch bodies and the statistical differences in U87B1-706A and fertile wheat. Students' *t* test *P<0.05 **P<0.01. Each value represents means ±SD (n=3).

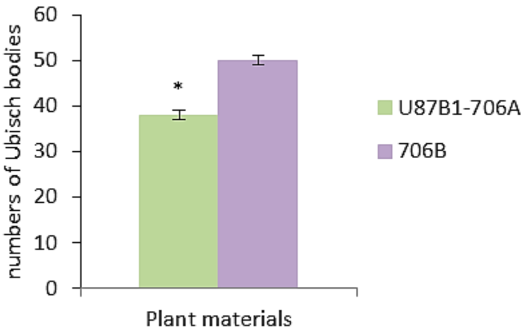

Supplementary Figure S2. Proportions of different abortion types in U87B1-706. Students' *t* test *P<0.05 **P<0.01. Each value represents means ±SD (n=3).

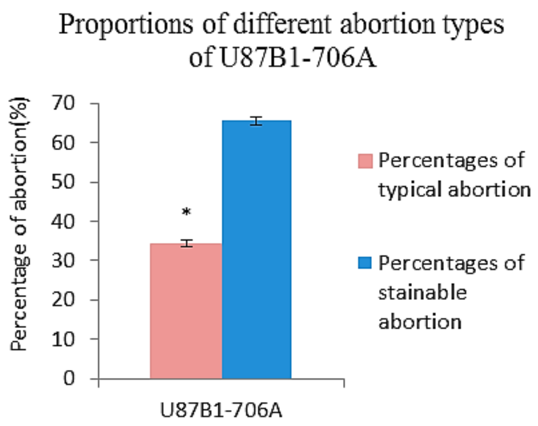

Supplementary Figure S3. Observations of the anther tapetum in U87B1-706A (A-E) and fertile wheat plants (F-J) during different developmental stages. (A, F) Td, tetrad stage; (B, G) Eun, early uninucleate stage; (C, H) Lun, late uninucleate stage; (D, I) Bn, binucleate stage; and (E, J) Tn, trinucleate stage. E, epidermis; En, endothecium; ML, middle layer; T, tapetum; Tds, tetrads; Msp, microspores. Scale bars are 50 µm in (A-J).

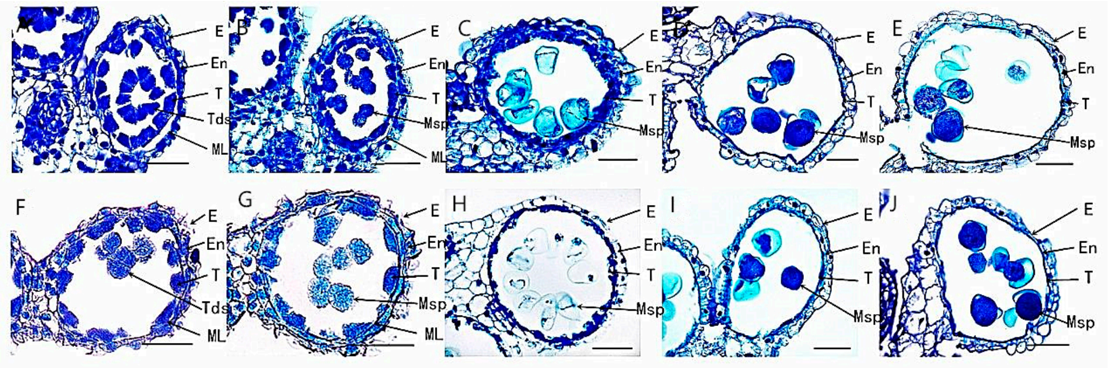

Supplementary Figure S4. Sectional area of the tapetum cell in U87B1-706A and fertile wheat. Students' *t* test *P<0.05 **P<0.01. Each value represents means ±SD (n=3).

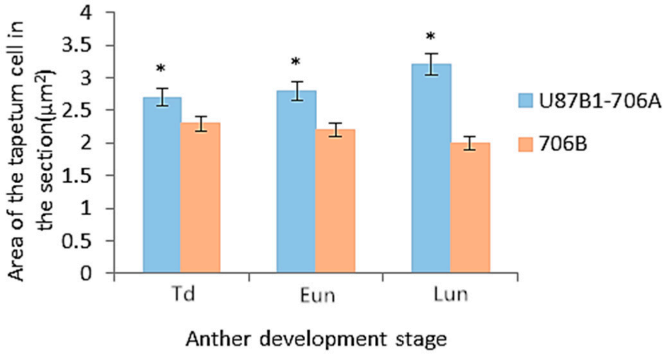

Supplementary Figure S5. TUNEL positive nuclei/total nuclei proportion for key stages.

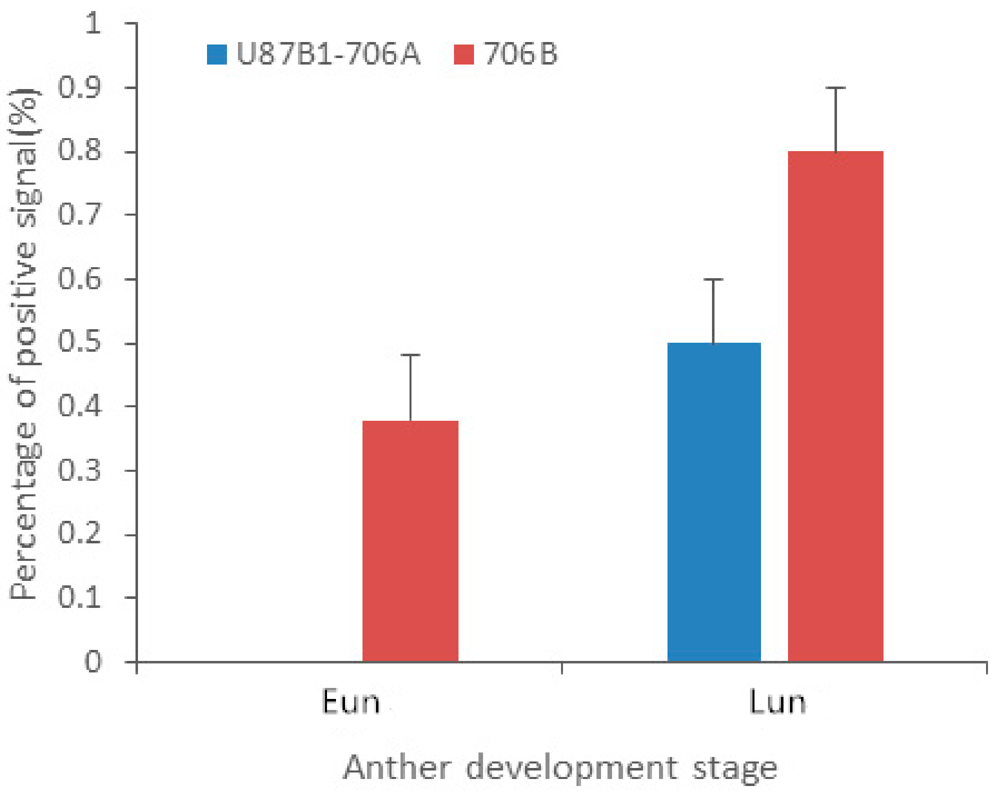

Supplementary Figure S6. Detection of DNA laddering in the anther tapetum from U87B1-706A and fertile wheat plants during different developmental stages. Td, tetrad stage; Eun, early uninucleate stage; Lun, late uninucleate stage; Bn, binucleate stage; and Tn, trinucleate stage. M, Marker D2000.

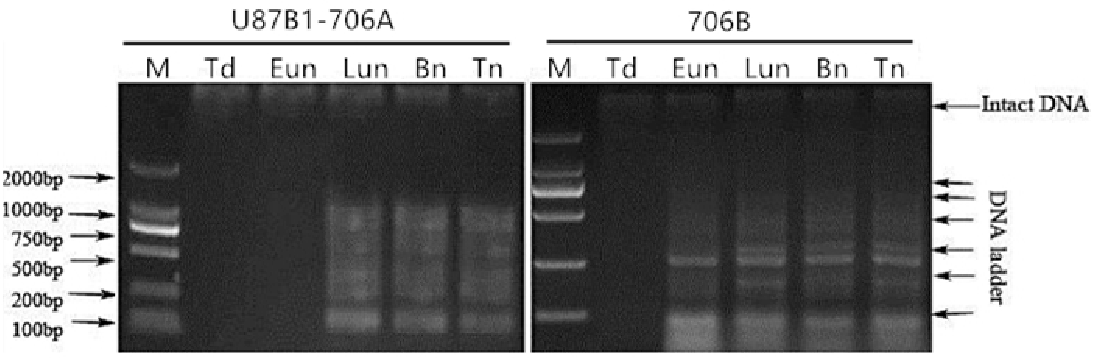

Supplementary Figure S7. Acetocarmine-stained of microspores in U87B1-706A (A-E) and 706B (F-J). (A, F) Td, tetrad stage; (B, G) Eun, early uninucleate stage; (C, H) Lun, late uninucleate stage; (D, I) Bn, binucleate stage; and (E, J) Tn, trinucleate stage. Scale bars are 50 μm in (A-J).

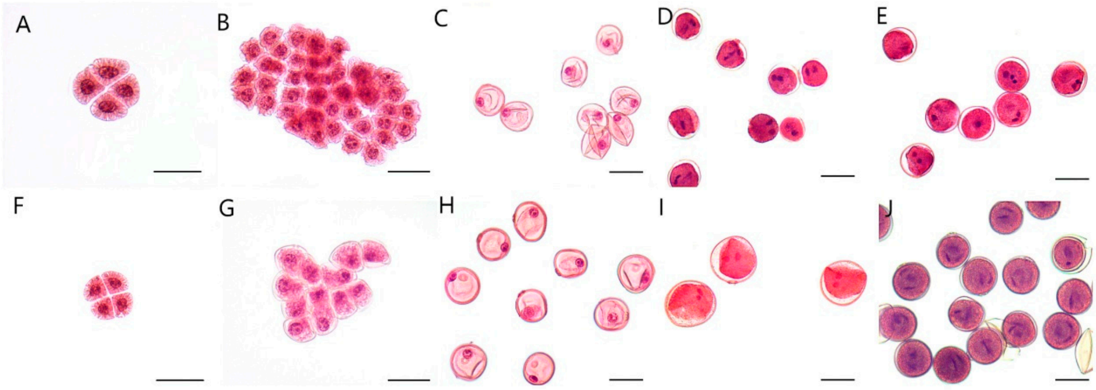

Supplementary Figure S8. Antioxidant enzyme genes detected by Reverse transcription-PCR in anthers during different development stages. Td, tetrad stage; Eun, early uninucleate stage; Lun, late uninucleate stage; Bn, binucleate stage; and Tn, trinucleate stage. M, Marker D2000; 1, U87B1-706A and 2,706B.

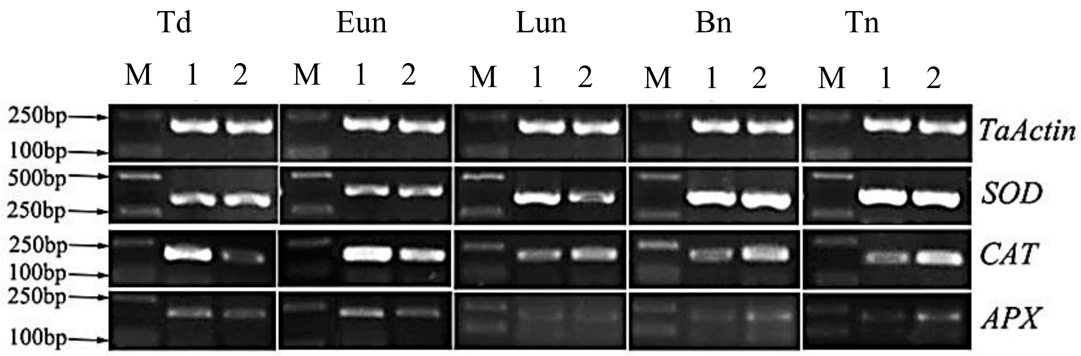

Supplementary Figure S9. Analysis of the correlations between the related enzyme genes (*SOD, CAT, APX*) expression levels and the enzyme (SOD, CAT, APX) activities in U87B1-706A and fertile wheat during different development stages. Td, tetrad stage; Eun, early uninucleate stage; Lun, late uninucleate stage; Bn, binucleate stage; and Tn, trinucleate stage.

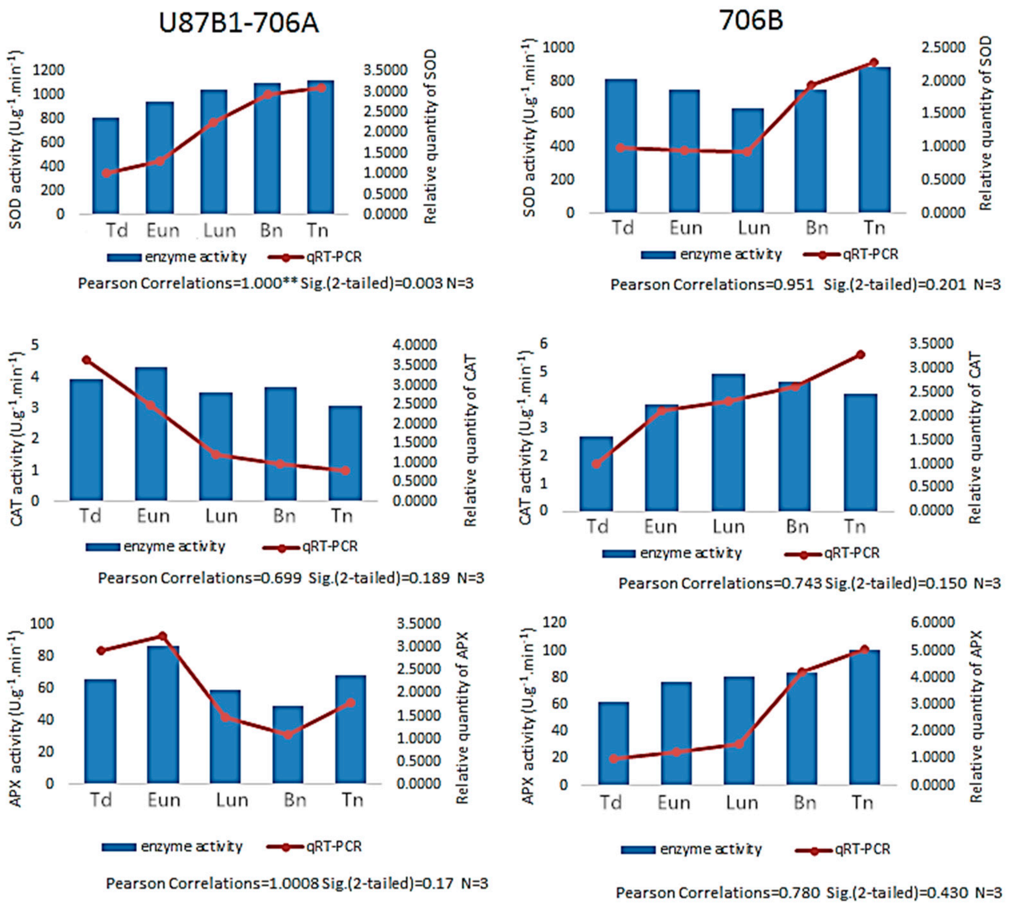

Supplement: Supplementary file 1 [file ijms-19-01708-s001.zip › ijms-313884-SI/Supplemental figures.pdf]
