# Supplementary material for: Tapetal-Delayed Programmed Cell Death (PCD) and Oxidative Stress-Induced Male Sterility of Aegilops uniaristata Cytoplasm in Wheat
Source: Int J Mol Sci. 2018 Jun 8;19(6):1708. doi: 10.3390/ijms19061708 (PMC6032135; doi:10.3390/ijms19061708)
Supplement: Supplementary file 1 [file ijms-19-01708-s001.zip › ijms-313884-SI/Supplemental table S1.pdf]

Supplementary Table S1 Primers used for quantitative real-time RT-PCR analysis antioxidant genes related to CMS

Primer	Sequence (5′-3′)	T_m/℃
SOD-f	AGAACCTCAAGCCTATCAGC	60
SOD-r	GACAAATCACGCAAGCACT	
CAT-f	TGCCTGTGTTTTTTATCCGA	62
CAT-r	ACCGTCCATGTGCCTGTAGT	
APX-f	GTTCATCCCTGGAAGACG	64
APX-r	CAGAGGGTCACGAGTCCA	
Actin-f	CTCCCTCACAACAACCGC	62
Actin-r	TACCAGGAACTTCCATACCAAC	
